# Alteration of Electron Acceptor Preferences in the Oxidative Half-Reaction of Flavin-Dependent Oxidases and Dehydrogenases

**DOI:** 10.3390/ijms21113797

**Published:** 2020-05-27

**Authors:** Kentaro Hiraka, Wakako Tsugawa, Koji Sode

**Affiliations:** 1 Department of Biotechnology and Life Science, Graduate School of Engineering, Tokyo University of Agriculture and Technology, 2-24-16 Naka-cho, Koganei, Tokyo 184-8588, Japan; s189868s@st.go.tuat.ac.jp (K.H.); tsugawa@cc.tuat.ac.jp (W.T.); 2 Joint Department of Biomedical Engineering, The University of North Carolina at Chapel Hill and North Carolina State University, Chapel Hill, NC 27599, USA

**Keywords:** oxidase, dehydrogenase, oxidative half-reaction, oxygen, electron acceptor, flavin adenine dinucleotide, flavin mononucleotide, oxygen accessible pathway, bioelectrochemistry, enzyme engineering

## Abstract

In this review, recent progress in the engineering of the oxidative half-reaction of flavin-dependent oxidases and dehydrogenases is discussed, considering their current and future applications in bioelectrochemical studies, such as for the development of biosensors and biofuel cells. There have been two approaches in the studies of oxidative half-reaction: engineering of the oxidative half-reaction with oxygen, and engineering of the preference for artificial electron acceptors. The challenges for engineering oxidative half-reactions with oxygen are further categorized into the following approaches: (1) mutation to the putative residues that compose the cavity where oxygen may be located, (2) investigation of the vicinities where the reaction with oxygen may take place, and (3) investigation of possible oxygen access routes to the isoalloxazine ring. Among these approaches, introducing a mutation at the oxygen access route to the isoalloxazine ring represents the most versatile and effective strategy. Studies to engineer the preference of artificial electron acceptors are categorized into three different approaches: (1) engineering of the charge at the residues around the substrate entrance, (2) engineering of a cavity in the vicinity of flavin, and (3) decreasing the glycosylation degree of enzymes. Among these approaches, altering the charge in the vicinity where the electron acceptor may be accessed will be most relevant.

## 1. Introduction

Oxidation reactions catalyzed by flavin-dependent oxidoreductases are widely used in areas such as the life sciences, chemistry, and industry. Flavin can receive two electrons from oxidizing substrates and release electrons to external electron acceptors in the oxidation reaction of flavin-dependent enzymes. Flavin-dependent oxidoreductases can be divided into oxidases, dehydrogenases, monooxygenases, halogenases, and so on [1]. Flavin-dependent oxidases and dehydrogenase based oxidation reactions, as well as their structural-function relationships, have been studied well in recent decades. Flavin-dependent oxidases oxidize substrates with the reduction of flavin (reductive half-reaction), and the reduced flavin is reoxidized by oxygen, liberating hydrogen peroxide (oxidative half-reaction). Flavin-dependent oxidases also utilize a variety of artificial electron acceptors instead of oxygen. The artificial electron acceptors refer to chemically synthesized redox active compounds, including organic, inorganic, and metal-organic compounds. A review article by Heller and Feldman well summarized the variation of artificial electron acceptors, by introducing them for glucose oxidoreductases [2]. However, in flavin-dependent oxidases, artificial electron acceptors compete with oxygen during the oxidative-half reaction [3,4,5]. Flavin-dependent dehydrogenases proceed with the same reductive half-reaction as that of oxidase, but they do not utilize oxygen as an electron acceptor in the oxidative half-reaction; instead, they use a variety of electron acceptors, including artificial electron acceptors [6,7].

In addition to the basic studies of reaction mechanisms, flavin-dependent oxidases and dehydrogenases have been utilized as molecular recognition elements of biosensors and biofuel cells in the electrochemical field. The first enzyme-based amperometric biosensor was reported in 1962 by Clark and Lyons using a combination of glucose oxidase and oxygen electrodes [8]. In the same year, the first enzymatic biofuel cell was also reported by Davis and Yarbrough using glucose oxidase as an anodic element [9]. Since then, many enzyme-based biosensors or biofuel cells have been developed [10,11]. After years of effort, flavin-dependent oxidoreductases, which include flavin mononucleotide (FMN)- and flavin adenine dinucleotide (FAD)-dependent oxidases and dehydrogenases, have been widely used to construct bioelectrochemical reaction based-biosensors and biofuel cells. Flavin-dependent oxidase-based biosensors detect the consumption of dissolved oxygen or the liberation of hydrogen peroxide depending on the substrate concentration, which is designated as the first-generation principle for electrochemical enzyme sensors [12]. However, dissolved oxygen fluctuation causes bias errors in the first-generation sensing system [13]. Further, a high applied potential for oxidizing hydrogen peroxide is required, which causes another bias error from the oxidation of interferents, such as ascorbic acid [14]. In addition, hydrogen peroxide potentially damages the electrode surface and the enzymes [15,16]. To overcome the inherent drawback of flavin-dependent oxidases, the use of artificial electron acceptors as electron mediators between the enzymes and the electrode has become popular, and the principle is designated as the second-generation principle, which was first reported in 1984 [14]. Moreover, various dehydrogenases have been utilized in second-generation principle-based sensors. However, the limited availability of flavin-dependent dehydrogenases is the key issue for the further development of second-generation enzyme sensors. The use of artificial electron acceptors with low oxidation potential helps to avoid the signals from redox active interferents during the measurements [2]. Considering the chemical stability, solubility, and redox potential, several electron acceptors were used to construct second-generation enzyme sensors using flavin-dependent oxidases and dehydrogenases [2,17]. However, each flavin-dependent oxidase or dehydrogenase has a preference for certain electron acceptors, and therefore, the improvement of specificities toward these mediators is in great demand [18,19].

In this review, recent progress in the investigation/engineering of the oxidative half-reaction of flavin-dependent oxidases and dehydrogenases is summarized and discussed, considering their current and future applications in bioelectrochemical studies, such as for the developments of biosensors and biofuel cells. These studies are categorized into two approaches: engineering of the oxidative half-reaction with oxygen, and engineering with the preference for artificial electron acceptors.

## 2. Engineering of the Oxidative Half-Reaction with Oxygen

**Figure 1:**
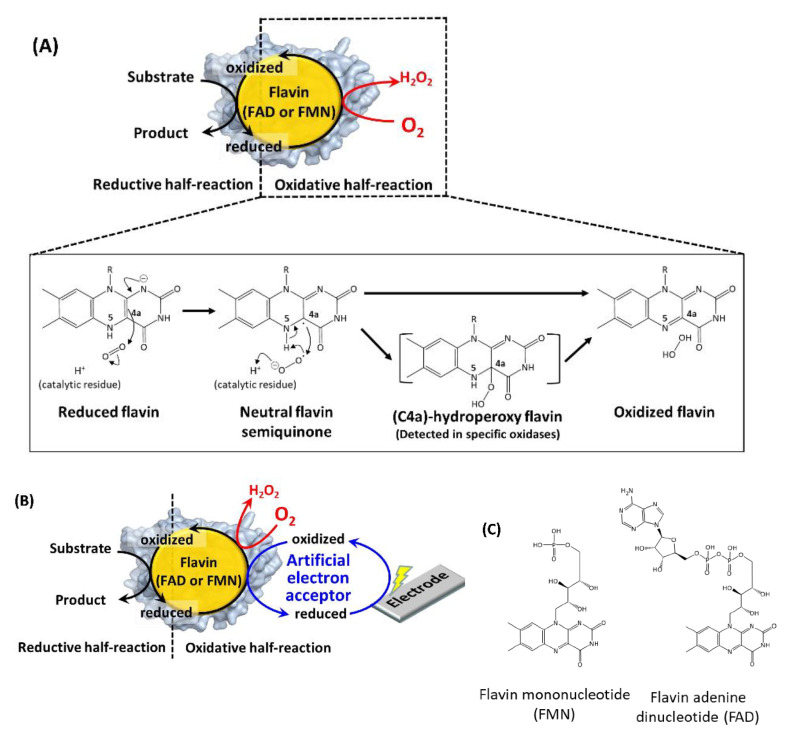
(**A**) Reaction scheme of a flavin-dependent oxidase with molecular oxygen [20]. (**B**) Reaction scheme of a flavin-dependent oxidase or dehydrogenase with an artificial electron acceptor. Oxidases react with oxygen, as shown by the red arrow and character. Both oxidases and dehydrogenases react with artificial electron acceptors as electron mediators between enzymes and electrodes. (**C**) Flavin mononucleotide (FMN) and flavin adenine dinucleotide (FAD) structural formulas.

Figure 1A explains the reaction scheme of the oxidases with molecular oxygen [20]. The reduced flavin transfers one electron to molecular oxygen to form a radical pair at isoalloxazine C4a in the first step. In the second step, another electron is transferred from isoalloxazine N5, or a protonated catalytic residue rapidly generates hydrogen peroxide and oxidized flavin [20,21]. The formation of (C4a)-hydroperoxy flavin was the common reaction intermediate in the oxidative half-reaction of flavin-dependent monooxygenases [1]. Except in some cases [22,23], this intermediate formation was not detected in flavin-dependent oxidases due to its rapid collapse, but it was considered a possible reaction intermediate [22]. The difference in the stability of this intermediate between oxidases and monooxygenases well explains the difference of the product formations in their oxidative half-reaction: hydrogen peroxide formation in oxidases, and inserting an oxygen atom into a substrate in monooxygenases [1,24]. Namely, the isoalloxazine C4a atom and its surrounding environment contributes to the reaction between oxygen and flavin, therefore, introducing mutations in the amino acid residues existing in this region are the representative rational strategy to engineer the oxidative half-reaction with oxygen in the flavin-dependent oxidases. However, it remains to be elucidated why and how “oxidases” can use oxygen as the primary electron acceptor in flavin-dependent oxidases, whereas “dehydrogenases” cannot, even though both oxidases and dehydrogenases proceed through identical reductive half-reactions with similar overall structures (e.g., glucose oxidase and glucose dehydrogenase) [25].

Pioneering studies reported the creation of “dehydrogenases” from oxidases to solve the inherent problem of oxidase-utilizing enzyme-based assays and electrochemical sensing systems employing artificial electron acceptors, where the competition of oxygen with artificial electron acceptors resulted in inaccurate results (Figure 1B).

**Figure 2:**
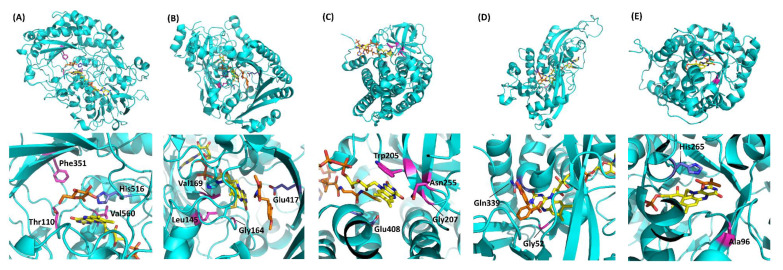
Representative flavin-dependent oxidase/dehydrogenase family enzyme structures. (**A**) AnGOx (PDB code: 1CF3), GMC family; (**B**) BBE (PDB code: 3D2D), VAO family; (**C**) Short-chain specific acyl-CoA oxidase (PDB code: 2IX5), ACO family; (**D**) DAO (PDB code: 1C0I), DAO family; (**E**) AvLOx (PDB code: 2E77), HAO family. The upper figures show the whole structure, and the lower figures show the flavin vicinity. Flavins are colored yellow, and substrate analogs are shown in orange. Purple amino acid residues are catalytic residues, and magenta residues are important residues for reactivity against oxygen. Gluconolactone in (A) was visualized by superimposition with AfGDH (PDB code: 4YNU) due to the high similarity (RMSD = 0.934 Å). Structure visualization was conducted using the PyMOL Molecular Graphics System, Version 2.2.3 Schrödinger, LLC.

Table 1 summarizes the representative studies reporting the investigation and engineering of the oxidative half-reaction of flavin-dependent oxidases and dehydrogenases with oxygen as the electron acceptor. These studies are categorized into three approaches: (1) mutation to the putative residues that compose the cavity where oxygen may be located, (2) investigation of the locations where the reaction with oxygen may take place, and (3) investigation of possible oxygen access routes to the isoalloxazine ring. These twelve oxidases and five dehydrogenases are classified into five enzyme families according to their characteristic structures (Figure 2) [3]. In this classification, four enzyme families are FAD-dependent enzymes and only one enzyme family is FMN-dependent enzymes. Due to the structural difference between FAD and FMN (Figure 1C), FAD-dependent enzymes have glycine rich motifs for binding with the adenine moiety of FAD, but FMN-dependent enzymes do not have a specific motifs for binding with FMN.

**Figure 3:**
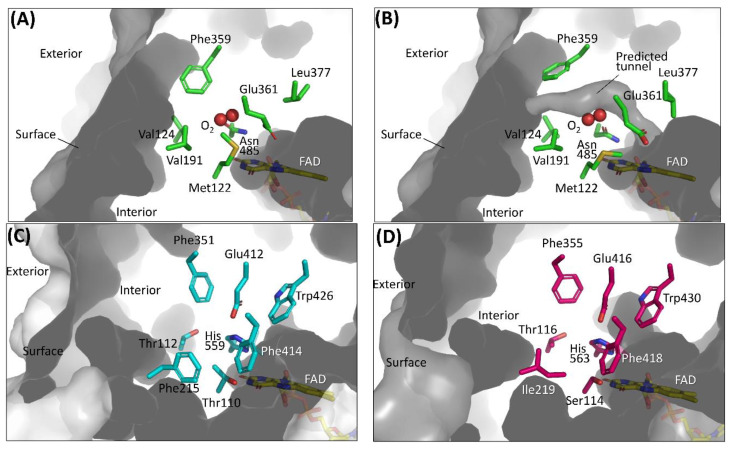
Amino acid residues composing the cavity where oxygen may be located in (**A**) ChOx (PDB code: 1MXT) conformation A, closed state; (**B**) ChOx (PDB code: 1MXT) conformation B, open state; (**C**) AnGOx (PDB code: 1CF3); (**D**) PaGOx (PDB code: 1GPE). Structure visualization was conducted using the PyMOL Molecular Graphics System, Version 2.2.3 Schrödinger, LLC. Tunnel visualization was performed for (A) and (B) by the PyMOL plugin CAVER 3.0.1. [51]. The starting point was FAD N5 and only the minimum probe radius was changed from the default value to 1.2 Å.

The first category focuses on the putative residues that are predicted to compose the cavity where oxygen may be located [26,27,28,29]. A sub-atomic resolution crystal structure of FAD-dependent cholesterol oxidase (ChOx) from *Streptomyces* sp. SA-COO was reported by Lario et al., providing a ChOx structure with a dioxygen molecule that was modeled in the active site [50]. Phe359, which is located proximate to the modeled oxygen molecule showed two conformations and opened or closed a hydrophobic tunnel from the external enzyme to the FAD vicinity depending on the Phe359 side chain orientation (Figure 3A,B). As the Phe359Trp mutant showed a drastic decrease in reactivity for oxygen, this hydrophobic tunnel was considered an oxygen-accessible pathway from an external enzyme to FAD [28]. However, the dye-mediated dehydrogenase activity of the Phe359Trp mutant was not investigated, and we could not know whether this mutation caused inactivation of the oxidative half-reaction. To reduce the reactivity for oxygen without loss of the dye-mediated dehydrogenase activity of ChOx, Kojima et al. reported mutations in amino acid residues that constitute the cavity where oxygen may interact, including Phe359 [29]. As a result, the Val191Ala mutant showed drastically low oxidase activity and increased dye-mediated dehydrogenase activity. Horaguchi et al. compared the active site structures of glucose oxidases (GOxs) and ChOx, considering that both oxidases are members of the glucose/methanol/choline (GMC) oxidoreductase family [26,27]. From the superposition of these structures, the putative cavity where oxygen may interact was predicted for GOx from *Penicillium amagasakiense* (PaGOx) and *Aspergillus niger* (AnGOx) (Figure 3C,D). Consequently, the PaGOx Ser114Ala and Phe355Leu, and AnGOx Thr110Ala and Phe351Leu mutations resulted in decreased oxidase activity when measuring hydrogen peroxide liberation, whereas the dye-mediated dehydrogenase activity increased. An enzyme electrode with a PaGOx Ser114Ala/Phe355Leu double mutant was constructed, and the impact of the presence or absence of oxygen during electron mediator-type enzyme sensor operation was investigated by comparing the electrode with wild-type PaGOx. The results revealed that the enzyme sensor with the PaGOx Ser114Ala/Phe355Leu double mutant showed a minimized impact of oxygen during measurement, specifically at a lower glucose concentration [27]. These results also suggested that GMC oxidoreductase family enzymes may have substrate- and oxygen-accessible tunnels, which are located on the Re-side of the isoalloxazine ring of flavin (Figure 2A).

The second category focuses on a cavity in the vicinity of the isoalloxazine ring [30,31,32,33,34,35,36,37,38,39,40,41,42,43,44,45]. Considering the reaction between oxygen and flavin, the region is likely crucial for the oxidative half-reaction. Based on crystal structural analyses, most of the approaches aimed to alter the oxidative half-reaction by mutating amino acid residues located near isoalloxazine ring C4a. Among them, Zafred et al. reported the study of oxygen reactivity in the vanillyl alcohol oxidase (VAO) superfamily by using the berberine bridge enzyme (BBE) and the BBE-like protein, pollen allergen Phl p 4, as models, showing that steric control of access to this site was the most important parameter affecting dioxygen reactivity in BBE-like enzymes [40]. BBE from *Eschsholzia californica* is (S)-reticuline oxidase, which is a member of the VAO superfamily, and Phl p 4 from *Phleum pratense* is also a VAO superfamily enzyme. BBE catalyzes (S)-reticuline oxidation with oxygen as a primary electron acceptor, but Phl p 4 catalyzes glucose oxidation without oxygen as an electron acceptor. BBE and Phl p 4 have similar structures, but the reactivity for oxygen is completely different. They focused on a difference in the cavity size in front of isoalloxazine C4a. BBE has a larger cavity than Phl p 4 in front of the FAD C4a, consisting of Gly164, Val169, and His174; but Phl p 4 has a smaller cavity than BBE, consisting of Gly148, Ile153, and Asn158. As a result of mutagenesis studies, the BBE Gly164Ala mutant showed a drastic decrease in the reaction rate constants with oxygen in the oxidative half-reaction (*k*_ox_), but the Phl p 4 Ile153Val mutant was increased by the *k*_ox_ value despite no change in the reaction rate constants for each substrate in the reductive half-reaction (*k*_red_). These mutations did not affect the redox potentials of these enzymes. Therefore, engineering of the cavity in front of isoalloxazine C4a enables control of the reactivity for oxygen. This report also revealed that substrates of VAO family enzymes come from the Si-side of the isoalloxazine ring, but an oxygen reaction occurred on the Re-side (Figure 2B). This reaction system is different from that of GMC oxidoreductase family enzymes.

Schwander et al. reported the conversion of (2*S*)-methylsuccinyl-CoA dehydrogenase (MCD) into oxidase and utilized it in the carbon dioxide fixation pathway in vitro to avoid the rate-limiting step of the crotonyl-CoA/ethylmalonyl-CoA/hydroxybutyryl-CoA (CETCH) cycle [44]. In this cycle, 17 enzyme reaction networks, including MCD, can convert carbon dioxide into glyoxylate; but MCD requires an external electron acceptor, such as ferrocenium, to achieve high carbon dioxide fixation efficiency. Therefore, the MCD reaction is the rate-limiting step of this cycle, and the authors tried to convert MCD into an oxidase. The MCD Trp315Phe/Thr317Gly/Glu377Asn mutant exhibited oxidase activity, and the CETCH cycle efficiency was increased without the addition of an artificial electron acceptor. A more detailed enzymatic study was reported by Burgener et al., who discussed that Thr317 of this enzyme played a key role as a dioxygen gatekeeper on the Si-side of the isoalloxazine ring [45]. As a result of site-directed mutagenesis of Thr317Gly, the oxidase activity was increased by removing the bulky amino acid side chain located on the considered oxygen reaction site. This enzyme is a member of the acyl-CoA oxidoreductase (ACO) family, and this family of enzymes accommodates the substrate on the Re-side of the isoalloxazine ring (Figure 2C). Considering the differences in the VAO and ACO family enzymes structures and oxygen uptake routes, the engineering of the cavity in the vicinity of the isoalloxazine ring is useful as a versatile method. Although structural information of the VAO and ACO family showing possible interaction with molecular oxygen is not currently available, the prediction of the structures with oxygen, based on the combination of homology modeling and computational simulation, will provide great perspectives for future engineering research [52].

In addition, Tremey et al. replaced the nonpolar Val residue near FAD of PaGOx with a polar Ser residue [30]. Although wild-type PaGOx showed differences in the *K*_m_ value for ferrocenemethanol (FM) in the presence or absence of oxygen due to the competition of oxygen and FM in the oxidative half-reaction of PaGOx, the Val564Ser mutant showed almost the same *K*_m_ value for FM in the same experiment in both the presence and absence of oxygen. PaGOx Val564Ser exhibited a decreased reactivity for oxygen at low concentrations of glucose and a mitigated oxygen interference effect on the electrode incorporated in an osmium polymer. This result indicated that the polarity alteration around flavin may change the reactivity for oxygen. However, Gutierrez et al. mutated the same position of AnGOx (Val560) in a random mutagenesis study prior to the work of Tremey et al. and succeeded in decreasing the activity for oxygen and increasing the reactivity for the mediator, and the authors discussed the perturbation of the catalytic hydrogen-bonding network [31]. These results indicated that engineering of a cavity in the vicinity of flavin alters the oxidative half-reaction of oxidase with oxygen.

The third category focuses on the oxygen-accessible pathway from external enzymes to the vicinity of flavin [46,47,48,49]. Saam et al. reported the study of D-amino acid oxidase (DAO) by identifying the oxygen diffusion channel from the solvent to the flavin and oxygen high-affinity sites around flavin using molecular dynamics simulations and implicit ligand sampling methods [47]. Gly52, which is located in an oxygen high-affinity site on the *Si*-side of flavin, was substituted with Val to occupy this site and to obstruct oxygen access. The DAO Gly52Val mutant showed a more than 100-fold decrease in the reactivity for oxygen, and without loss of catalytic reactivity in the reductive half-reaction. Since the substrate was recognized and oxidized on the Re-side of the isoalloxazine ring, a proton relay system based on a hydrogen-bonding network between amino acids around FAD was proposed. This system relayed protons from the substrate on the Re-side to oxygen on the Si-side of FAD; thus, the Gly52Val mutant was considered to disrupt the proton relay system in DAO (Figure 2D). Kim et al. reported the engineering of fructosyl amino acid oxidase (FAOx) from *Pichia* sp. N1-1 and amadoriase II from *Aspergillus fumigatus* to construct enzymes with decreased oxidase activity while retaining dye-mediated dehydrogenase activity [37]. The authors introduced mutations at residues composing the putative proton relay system of FAOx, focusing on the structural similarity of FAOx and DAO [47]. As a result, the FAOx Asn47Ala mutant and amadoriase II Asn52Ala mutant showed a drastic decrease in oxidase activity and maintained dye-mediated dehydrogenase activity. The authors also reported the engineering of fructosyl peptidyl oxidase (FPOx) from *Phaeosphaeria nodorum*. Compared with the wild-type, the FPOx Asn56Ala mutant showed an oxidase activity *V*_max_ value of 18% and a dye-mediated dehydrogenase activity *V*_max_ value of 233% [38]. The FPOx Asn56Ala mutant decreased the oxygen interference effect on the electrochemical system with an artificial electron mediator compared with the wild-type. Shimasaki et al. reported the X-ray structures of FPOx wild-type and Asn56Ala mutant [53], revealing that the main reason for the alteration of electron acceptor preference toward oxygen was due to the formation of novel hydrogen bonds which might eliminate the accessibility of oxygen toward the short pathway, thereby resulting in a drastic decrease in oxidase activity compared with dye-mediated dehydrogenase activity.

**Figure 4:**
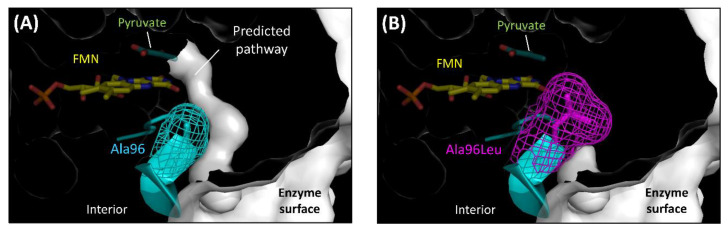
Obstruction of the predicted oxygen-accessible pathway in the AvLOx structure. (**A**) Wild type LOx from *Aerococcus viridans*. (PDB code: 2E77) (**B**) AvLOx Ala96Leu mutant. Structure visualization and mutation were conducted using the PyMOL Molecular Graphics System, Version 2.2.3 Schrödinger, LLC and its mutagenesis wizard. Tunnel visualization was performed by the PyMOL plugin CAVER 3.0.1. [51], the starting point was FMN C4a, and only the minimum probe radius was changed from default value to 0.85 Å.

Hiraka et al. predicted the oxygen-accessible pathway in lactate oxidase (LOx), an FMN oxidoreductase, and attempted to block this pathway by amino acid substitution to decrease the oxidative half-reaction with oxygen (Figure 4) [48,49]. *Aerococcus viridans* LOx (AvLOx) mutants decreased oxidase activity drastically (only 1.0% that of wild-type) but retained dye-mediated dehydrogenase activity (110% that of wild type) without any change in the substrate specificity and thermal stability. The AvLOx Ala96Leu mutant also mitigated the effect of oxygen interference on the electrochemical enzyme sensor employing an artificial electron acceptor. This was the first case of altering the reactivity for oxygen in the oxidative half-reaction in an α-hydroxy acid oxidoreductase (HAO) family, which is an FMN-dependent enzyme and has a (β/α)_8_ TIM barrel fold, a completely different conformation to that of FAD-dependent enzymes (Figure 2E).

These studies suggested that the third category, engineering of oxygen-accessible pathways, will be available for several family enzymes to engineer the reactivity for oxygen.

## 3. Engineering of the Preference for Artificial Electron Acceptors

Flavin-dependent oxidases and dehydrogenases utilize a variety of artificially synthesized electron acceptors, such as organic (e.g., quinone, viologen, and phenazine derivatives), inorganic (e.g., complex of iron, cobalt, and ruthenium), and metal-organic (e.g., osmium polymers and ferrocene polymers) compounds [2]. In the commercially available enzyme sensors for glucose, lactate and cholesterol, and in academic research about biosensors and biofuel cells, varieties of artificial electron acceptors were used as mediators between flavin-dependent oxidases/dehydrogenases and electrodes. Considering the practical application of biosensors and biofuel cells, artificial electron acceptors with high stability and with low redox potential have been preferably utilized, such as hexaammineruthenium and naphthoquinone [54,55]. However, the availability of artificial electron acceptors depends on the type of flavin-dependent oxidase or dehydrogenase, which demonstrate electron acceptor preferences. Therefore, the improvement of specificities or sensitivities toward these artificial electron acceptors is strongly desired. Pioneering research by Dixon summarized the electron acceptor specificity of flavins and flavoproteins in 1971 [56,57,58]. Although free reduced flavin can utilize and is oxidized by all the commonly used electron acceptors, the reduced flavin bound in proteins showed electron acceptor specificities depending on the proteins. With increasing demand for the use of varieties of artificial electron acceptors in electrochemical enzyme sensors, alteration of the electron acceptor preferences of flavin-dependent oxidoreductases are also increasing. Table 2 summarizes the representative studies reporting the investigation and engineering of artificial electron acceptor preferences. Four flavin-dependent oxidases and two dehydrogenases were reported, and their preference toward electron acceptors was changed by amino acid substitutions. These studies are categorized into three different approaches: (1) engineering of the charge at the residues around the substrate entrance, (2) engineering of a cavity in the vicinity of flavin, and (3) decreasing the glycosylation degree of enzymes.

**Figure 5:**
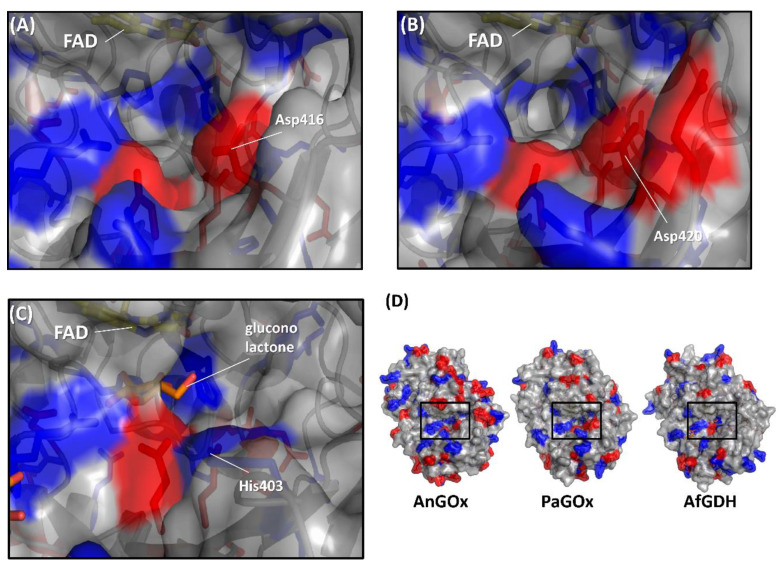
Comparison of the surface charge around the substrate entrance of (**A**) AnGOx (PDB code: 1CF3), (**B**) PaGOx (PDB code: 1GPE), and (**C**) AfGDH (PDB code: 4YNU). (**D**) Whole structure of these enzymes, and black squares show the substrate entrance. The red colored region shows the negatively charged residue, and the blue region shows the positively charged residue. Structure visualization was conducted using the PyMOL Molecular Graphics System, Version 2.2.3 Schrödinger, LLC.

The first category was specifically focused on the charge proximal to the substrate entrance pocket of enzymes belonging to the GMC oxidoreductase family, considering that the charge of artificial electron acceptors can possibly result in electrostatic repulsion with enzymes [58,59]. Suraniti et al. attempted to improve the current densities of enzymatic anodes constructed with PaGOx and an osmium polymer [60]. The osmium complex is a positively charged redox electron acceptor. Therefore, the authors reported the substitution of a negatively charged amino acid residue into a positively charged amino acid residue positioned around the substrate entrance. As a result, the PaGOx Lys424Glu mutant showed a 2.4-fold higher current density than wild-type PaGOx, when osmium polymer was used as the electron acceptor. This research group combined the PaGOx Lys424Glu mutant and Val564Ser mutant to obtain a higher response current density without an oxygen interference effect [30]. Okurita et al. reported an alteration of the preference toward the positively charged electron acceptor, hexaammineruthenium(III), of glucose dehydrogenase derived from *Aspergillus flavus* (AfGDH) and AnGOx [59]. AfGDH could not utilize hexaammineruthenium(III) as the electron acceptor, whereas AnGOx could. Comparing the 3D structures of AfGDH and AnGOx, AnGOx has a negatively charged amino acid (Asp416) on the substrate entrance pocket, but AfGDH has a positively charged amino acid (His430), which corresponds to Asp416 of AnGOx (Figure 5). A single amino acid substitution of AfGDH, His430Asp and His430Glu, showed a drastic increase in enzyme activity using hexaammineruthenium(III) as the electron acceptor. In contrast, AnGOx Asp416His or Asp416Ala amino acid substitution almost eliminated enzyme activity using hexaammineruthenium(III) as the electron acceptor. These results demonstrated that charged residues around the substrate entrance pocket were responsible for determining the electron acceptor preference.

The second category focused on the amino acid residue in the vicinity of flavin [60,61,62]. Yorita et al. focused on AvLOx and planned to convert it into lactate monooxygenase (LMO). LOx Ala95, which is located in front of FMN N5, was substituted for Gly, considering that the corresponding residue of LMO was Gly [65]. However, AvLOx Ala95Gly was not turned into LMO, but the substrate specificity was changed from L-lactate to long-chain α-hydroxy acids instead. Stoisser et al. reinvestigated the AvLOx Ala95Gly mutant, revealing that the mutation affected not only reductive half-reaction but also oxidative half-reaction [62]. LOx Ala95Gly mutation changed *k*_ox_ values with various artificial electron acceptors, which was not dependent on redox potential of the acceptors. The authors concluded that the LOx Ala95Gly mutant removed the steric bulk of the alanine side chain and caused a change in the electron acceptor specificity. This result also suggested that the electron acceptors enter the flavin vicinity as well as the substrate.

The third category focused on the correlation of glycosylation and enzyme electron acceptor preferences. The representative approach is the removal of glycosylation moieties of glycosylated flavin-dependent oxidases or dehydrogenases by chemical or endoglycosidase treatment. The deglycosylated GOx [66], GDH [67], PDH [68,69,70], and CDH [71] were immobilized on electrodes with osmium polymers and showed higher current density than glycosylated enzymes after addition of each substrate. The removal of the glycan shell contributed to the access of the redox polymer toward the active site of enzymes. However, these deglycosylation treatments are time-consuming and costly [63]. An alternative approach was performed with pyranose dehydrogenase (PDH) from *Agaricus meleagris* which is a glycosylated enzyme. The PDH crystal structure was analyzed [72], and the N-glycosylated site was also determined [68]. The non-glycosylated mutants of PDH were investigated for their electrochemical properties, compared with those with glycosylation using osmium polymer as the electron mediator. Yakovleva et al. reported that a number of *A. meleagris* PDHs, exhibiting different degrees of glycosylation, were produced using site-directed mutagenesis and electrochemically characterized [63]. They prepared Asn75Gly, Asn175Gln, Asn75Gly/Asn175Gln, and Asn75Gly/Asn175Gln/Asn252Gln PDH mutants. These mutants lacked glycosylated asparagine (N) residues and were expressed as enzymes in *Pichia pastoris* that lack part of the glycosyl moiety. Enzyme electrodes with PDH mutants were constructed together with various osmium polymers. As a result of flow-injection amperometry analyses, the Asn75Gly/Asn175Gln double mutant showed the highest current density using Os(dmbpy)-PVI and Os(bpy)-PVI; the Asn75Gly/Asn175Gln/Asn252Gln triple mutant indicated the highest current density with Os(dmobpy)-PVI. These results demonstrated that PDH mutants with fewer glycosylation moieties showed higher electron transfer efficiency with external electron acceptors, probably because the active site was more accessible to the osmium polymer, and electron transfer became faster. Although less glycosylated PDH mutants showed lower thermal stability due to the decreased glycosylation degree [64], the mutagenesis approach seems to be more practical than using deglycosylation treatments for alteration of the electron acceptor preference of glycosylated enzymes.

## 4. Conclusion

With increasing demands for the use of flavin-dependent oxidases and dehydrogenases for electrochemical applications, the investigation and engineering of the oxidative half-reaction of these enzymes have been increasing in significance. There have been two approaches in the studies of the oxidative half-reaction of flavin-dependent oxidases and dehydrogenases: engineering of the oxidative half-reaction with oxygen, and engineering of the preference for artificial electron acceptors.

Pioneering challenges have been reported to create dehydrogenases from oxidases to solve the inherent problem of oxidase-utilizing enzyme-based assays and electrochemical sensing systems employing artificial electron acceptors, where the competition of oxygen and artificial electron acceptors resulted in the inaccurate results. These challenges to engineering oxidative half-reactions with oxygen are categorized into the following approaches: (1) mutation to the putative residues that compose the cavity where oxygen may be located, (2) investigation of the locations where the reaction with oxygen may take place, and (3) investigation of possible oxygen access routes to the isoalloxazine ring.

The demand for the use of a variety of artificial electron acceptors in electrochemical enzyme sensors triggered research to alter the preference of electron acceptors of flavin-dependent oxidoreductases. The studies to engineer the preference of artificial electron acceptors are categorized into three different approaches: (1) engineering of the charge at the residues around the substrate entrance, (2) engineering of a cavity in the vicinity of flavin, and (3) decreasing the glycosylation degree of enzymes.

Among these approaches, introducing a mutation to the possible oxygen access route to the isoalloxazine ring represents the most versatile and effective strategy to create dehydrogenase from oxidase. To alter the preference of artificial electron acceptors, changing the charge in the vicinity where the electron acceptor is accessible will be most relevant, considering the charge of electron acceptors.

Alteration of electron acceptor preferences of flavin-dependent oxidases and dehydrogenases is an attractive approach in enzyme engineering, not only to elucidate the important amino acid residues for recognizing electron acceptors, but also to extend the future potential applications of existing well-characterized enzymes, such as GOx and LOx, for novel applications and transducers being developed to achieve high performance bioelectrochemical devices.

## Figures and Tables

**Table 1:** The investigation/engineering the oxidative half-reaction of flavin-dependent oxidases and dehydrogenases with oxygen as the electron acceptor.

Enzyme (Source)	Family (EC: Number)	Co-Factor	Mutation Site	Mutated Position	Effects of Mutation	Reference
**(1) Investigation/engineering of the putative residues which are predicted oxygen binding site**						
Glucose oxidase (*Penicillium amagasakiense*)	GMC (EC: 1.1.3.16)	FAD	S114A, F355L	Predicted oxygen binding site	Decreased oxidase activity	[26,27]
Glucose oxidase (*Aspergillus niger*)	GMC (EC: 1.1.3.16)	FAD	T110A, F351L	Predicted oxygen binding site	Decreased oxidase activity	[26]
Cholesterol oxidase (*Streptomyces* sp. SA-COO)	GMC (EC: 1.1.3.6)	FAD	V191A, F359W	Oxygen binding site of crystal structure	Decreased oxidase activity	[28,29]
**(2) Investigation/engineering of the cavity in vicinity of isoalloxazine ring**						
Glucose oxidase (*Penicillium amagasakiense*)	GMC (EC: 1.1.3.16)	FAD	V564S	Vicinity of FAD	Decreased oxidase activity	[30]
Glucose oxidase (*Aspergillus niger*)	GMC (EC: 1.1.3.16)	FAD	V560T (random mutation)	Vicinity of FAD	Decreased oxidase activity	[31]
Pyranose oxidase (*Trametes multicolor*)	GMC (EC: 1.1.3.10)	FAD (covalent)	L547R, N593C	Vicinity of FAD	Decreased oxidase activity	[32,33]
Choline oxidase (*Arthrobacter globiformis*)	GMC (EC: 1.1.3.17)	FAD (covalent)	V464T, V464A	Vicinity of FAD	Decreased oxidase activity	[34]
Aryl-alcohol oxidase (*Pleurotus eryngii*)	GMC (EC: 1.1.3.7)	FAD	F501A, F397W	Vicinity of FAD	Decreased oxidase activity	[35,36]
			F501W	Vicinity of FAD	Increased oxidase activity	[35]
Fructosyl amino acid oxidase (*Pichia* sp. N1-1)	DAO (EC: 1.5.3)	FAD (covalent)	K276M, N47A	Proton relay system (Vicinity of FAD)	Decreased oxidase activity	[37]
Fructosyl peptide oxidase (*Phaeosphaeria nodorum*)	DAO (EC: 1.5.3)	FAD (covalent)	N56A	Proton relay system (Vicinity of FAD)	Decreased oxidase activity	[38]
Monomeric sarcosine oxidase (*Bacillus* sp. B-0618)	DAO (EC: 1.5.3.1)	FAD (covalent)	K265M	Proton relay system (Vicinity of FAD)	Decreased oxidase activity	[39]
Berberine bridge enzyme (Reticuline oxidase) (*Eschscholzia californica*)	VAO (EC: 1.21.3.3)	FAD (covalent)	G164A, V169I	Vicinity of FAD	Decreased oxidase activity	[40]
Cellobiose dehydrogenase (*Myriococcum thermophilum*)	GMC (EC: 1.1.99.18)	FAD	N700S	Vicinity of FAD	Increased oxidase activity	[41]
Pyranose dehydrogenase (*Agaricus meleagris*)	GMC (EC: 1.1.99.29)	FAD (covalent)	H103Y	Vicinity of FAD (Covalent bond with FAD)	Breaking of covalent bond with FAD Increased oxidase activity	[42]
L-Galactono-γ-lactone dehydrogenase (*Arabidopsis thaliana*)	VAO (EC: 1.3.2.3)	FAD (covalent)	A113G	Vicinity of FAD	Increased oxidase reactivity	[43]
Pollen allergen Phl p 4 (*Phleum pretense*)	VAO (EC: -)	FAD (covalent)	I153V	Vicinity of FAD	Increased oxidase activity	[40]
(2*S*)-methylsuccinyl-CoA dehydrogenase (*Rhodobacter sphaeroides*)	ACO (EC: 1.3.8.12)	FAD	W315F/T317G/E377N	Vicinity of FAD	Increased oxidase activity	[44,45]
**(3) Investigation/engineering of possible oxygen access route from external enzyme to the isoalloxazine ring**						
Choline oxidase (*Arthrobacter globiformis*)	GMC (EC: 1.1.3.17)	FAD (covalent)	F357A	Predicted oxygen accessible pathway	Decreased oxidase activity	[46]
D-amino acid oxidase (*Rhodotorula gracilis*)	DAO (EC: 1.4.3.3)	FAD	G52V	Predicted oxygen accessible pathway	Decreased oxidase activity	[47]
L-lactate oxidase (*Aerococcus viridans*)	HAO (EC: 1.1.3.15)	FMN	A96L, N212K, A96L/N212K	Predicted oxygen accessible pathway	Decreased oxidase activity	[48,49]

**Table 2:** Engineering approaches to alter the preferences of flavin-dependent oxidases and dehydrogenases for artificial electron acceptors.

Enzyme (Source)	Family (EC: Number)	Co-Factor	Mutation Site	Mutated Position	Effects of Mutation	Reference
**(1) Engineering of surface residue around the substrate entrance**						
Glucose oxidase (*Penicillium amagasakiense*)	GMC (EC: 1.1.3.16)	FAD	K424E	Surface charged residue	Increased reactivity for osmium polymer	[59]
Glucose oxidase (*Aspergillus niger*)	GMC (EC: 1.1.3.16)	FAD	D416A, D416H	Surface charged residue	Decreased reactivity for hexaammineruthenium (III)	[55]
Glucose dehydrogenase *(Aspergillus flavus)*	GMC (EC: 1.1.5.9)	FAD	H403D	Surface charged residue	Increased reactivity for hexaammineruthenium (III)	[55]
**(2) Engineering of a cavity in the vicinity of flavin**						
Glucose oxidase V7 mutant *(Aspergillus niger)*	GMC (EC: 1.1.3.16)	FAD	I414M, I414Y	Vicinity of FAD	Increased reactivity for quinone diamine based electron acceptors	[60]
Cytokinin oxidase *(Zea mays)*	VAO (EC: 1.5.99.12)	FAD (covalent)	D169E, L492A	Vicinity of FAD	Increased reactivity for several artificial electron acceptors	[61]
L-lactate oxidase *(Aerococcus viridans)*	HAO (EC: 1.1.3.15)	FMN	A95G	Vicinity of FMN	artificial electron acceptor Decreased substrate specificity	[62]
**(3) Decreasing the glycosylation degree of the enzyme**						
Pyranose dehydrogenase *(Agaricus meleagris)*	GMC (EC: 1.1.99.29)	FAD (covalent)	N75G, N175Q, N252Q	Glycosylation site	Decreased glycosylation degree Increased reactivity for osmium polymer Decreased thermal stability	[63,64]

## Abbreviations

ACO	Acyl-CoA oxidase
AfGDH	Glucose dehydrogenase from *Aspergillus flavus*
AnGOx	Glucose oxidase from *Aspergillus niger*
AvLOx	L-lactate oxidase from *Aerococcus viridans*
BBE	berberine bridge enzyme
ChOx	Cholesterol oxidase
DAO	D-amino acid oxidase
DET	Direct electron transfer
FAD	Flavin adenine dinucleotide
FAOx	Fructosyl amino acid oxidase
FM	Ferrocene methanol
FMN	Flavin mononucleotide
FPOx	Fructosyl peptide oxidase
GMC	Glucose-methanol-choline
GOx	Glucose oxidase
HAO	α-hydroxy acid oxidase
LMO	Lactate monooxygenase
LOx	L-lactate oxidase
MCD	(2*S*)-methylsuccinyl-CoA dehydrogenase
PDB	Protein data bank
PDH	Pyranose 2-dehydrogenase
PaGOx	Glucose oxidase from Penicillium amagasakiense
VAO	Vanillyl alcohol oxidase
